# *Spiroplasma* as facultative bacterial symbionts of stinkbugs

**DOI:** 10.3389/fmicb.2022.1044771

**Published:** 2022-10-24

**Authors:** Shigeyuki Kakizawa, Takahiro Hosokawa, Kohei Oguchi, Kaori Miyakoshi, Takema Fukatsu

**Affiliations:** ^1^Bioproduction Research Institute, National Institute of Advanced Industrial Science and Technology (AIST), Tsukuba, Japan; ^2^Department of Biology, Faculty of Science, Kyushu University, Fukuoka, Japan; ^3^Misaki Marine Biological Station (MMBS), School of Science, The University of Tokyo, Miura, Japan; ^4^Department of Biological Sciences, Graduate School of Science, The University of Tokyo, Tokyo, Japan; ^5^Graduate School of Life and Environmental Sciences, University of Tsukuba, Tsukuba, Japan

**Keywords:** *Spiroplasma*, symbiosis, bacteria, stinkbug, Hemiptera, Pentatomidae

## Abstract

Many insects are associated with facultative symbiotic bacteria, and their infection prevalence provides an important clue to understand the biological impact of such microbial associates. Here we surveyed diverse stinkbugs representing 13 families, 69 genera, 97 species and 468 individuals for *Spiroplasma* infection. Diagnostic PCR detection revealed that 4 families (30.8%), 7 genera (10.1%), 11 species (11.3%) and 21 individuals (4.5%) were *Spiroplasma* positive. All the 21 stinkbug samples with *Spiroplasma* infection were subjected to PCR amplification and sequencing of *Spiroplasma*’s 16S rRNA gene. Molecular phylogenetic analysis uncovered that the stinkbug-associated *Spiroplasma* symbionts were placed in three distinct clades in the Spiroplasmataceae, highlighting multiple evolutionary origins of the stinkbug-*Spiroplasma* associations. The *Spiroplasma* phylogeny did not reflect the host stinkbug phylogeny, indicating the absence of host-symbiont co-speciation. On the other hand, the *Spiroplasma* symbionts associated with the same stinkbug family tended to be related to each other, suggesting the possibility of certain levels of host-symbiont specificity and/or ecological symbiont sharing. Amplicon sequencing analysis targeting bacterial 16S rRNA gene, FISH visualization of the symbiotic bacteria, and rearing experiments of the host stinkbugs uncovered that the *Spiroplasma* symbionts are generally much less abundant in comparison with the primary gut symbiotic bacteria, localized to various tissues and organs at relatively low densities, and vertically transmitted to the offspring. On the basis of these results, we conclude that the *Spiroplasma* symbionts are, in general, facultative bacterial associates of low infection prevalence that are not essential but rather commensalistic for the host stinkbugs, like the *Spiroplasma* symbionts of fruit flies and aphids, although their impact on the host phenotypes should be evaluated in future studies.

## Introduction

Diverse insects are generally in symbiotic association with microorganisms (Buchner, 1965; Bourtzis and Miller, 2003). Some symbionts are obligatory microbial partners essential for their hosts *via* helping digestion, supplementing essential nutrients, or undertaking other important biological roles (Moran et al., 2008; Douglas, 2009; Brune, 2014). Other symbionts are facultative microbial associates affecting their hosts either positively *via* conferring context-dependent benefits such as defense against natural enemies, resistance against parasites and pathogens, tolerance against abiotic stressors, etc. (Flórez et al., 2015; Van Arnam et al., 2018; Lemoine et al., 2020), neutrally with no apparent phenotypic consequences, or negatively *via* reducing host growth, survival and/or fecundity in a variety of ways (Oliver et al., 2010; Feldhaar, 2011).

Members of the alphaproteobacterial genus *Wolbachia* are known as the most widespread facultative symbiotic bacteria of insects and other arthropods (Werren et al., 2008; Kaur et al., 2021). While *Wolbachia* symbionts are famous for their capabilities of manipulating host reproduction by inducing cytoplasmic incompatibility, parthenogenesis, feminization or male-killing (Werren et al., 2008; Kaur et al., 2021), which generally affect the host fitness negatively, some *Wolbachia* strains were reported to entail positive fitness consequences *via* conferring resistance to pathogen infections (Hedges et al., 2008; Teixeira et al., 2008), supplying essential nutrients (Hosokawa et al., 2010; Nikoh et al., 2014; Moriyama et al., 2015), or slightly enhancing growth and/or fecundity often in a context-dependent manner (Zug and Hammerstein, 2015). In addition, the diverse insects may also host such facultative symbiotic bacteria as *Rickettsia*, *Cardinium*, *Sodalis*, *Arsenophonus*, *Spiroplasma*, etc. (Zchori-Fein and Perlman, 2004; Perlman et al., 2006; Duron et al., 2008; Nováková et al., 2009; Hosokawa et al., 2015). In comparison with the extensively compiled data on the infection prevalence and diversity of *Wolbachia* in natural host species and populations (ex. Zug and Hammerstein, 2012; Gerth et al., 2014), it has been less explored and still to be established how these facultative microbial associates other than *Wolbachia* are prevalent in the natural host diversity (Duron et al., 2008).

Members of the bacterial genus *Spiroplasma*, belonging to the class Mollicutes of the phylum Tenericutes (or Mycoplasmatota as recently proposed; see Oren and Garrity, 2021), are, typically, helical in shape, actively motile, and lacking outer cell wall (Gasparich et al., 2020). Initially, they were described as plant disease agents *S. kunkeli* for corn stunt disease (Davis et al., 1972) and *S. citri* for citrus stubborn disease (Saglio et al., 1973), then identified as male-killing sex ratio distorters of fruit flies (Poulson and Sakaguchi, 1961) later named *S. poulsonii* (Williamson et al., 1999), and thus far recognized as widely associated with diverse insects and other terrestrial arthropods (Clark, 1982), and more recently, also found from aquatic invertebrates including crustaceans, sea cucumbers and jellyfish (Nunan et al., 2005; Wang et al., 2005, 2011; He et al., 2017; Viver et al., 2017). Of the diverse *Spiroplasma* species and strains, some were reported as either highly or weakly pathogenic to plants, insects and crustaceans (Daniels, 1983; Fukatsu et al., 2001; Wang et al., 2005; Simon et al., 2011), some were shown to provide their hosts with defensive benefit against parasitic wasps, nematodes and fungi (Jaenike et al., 2010; Xie et al., 2010; Łukasik et al., 2013; Xie et al., 2014), some were noted for causing prominent male-killing phenotypes in flies, lady beetles, butterflies, moths, planthoppers and aphids (Jiggins et al., 2000; Hurst et al., 2003; Haselkorn, 2010; Simon et al., 2011; Sanada-Morimura et al., 2013), but the remaining majority are recognized solely by PCR detection and/or sequencing of bacterial gene fragments, with little biological information being available (Anbutsu and Fukatsu, 2011; Ballinger and Perlman, 2019).

In this context, stinkbugs (Hemiptera: Pentatomoidea) represent a notable insect group in that their obligatory and facultative symbiotic bacteria have been well surveyed at species and population levels (Kikuchi and Fukatsu, 2003; Matsuura et al., 2012; Hosokawa et al., 2015, 2016). The majority of plant-sucking stinkbugs develop a midgut symbiotic organ consisting of an assemblage of numerous sac- or tube-like crypts, whose inner cavities harbor a dense population of specific symbiotic bacteria (Buchner, 1965; Salem et al., 2015; Takeshita and Kikuchi, 2017). These gut symbionts are generally indispensable for normal growth, survival and/or reproduction of the host stinkbugs, being mutualistic microbial associates whose biological roles are presumed as provisioning of essential amino acids, vitamins and other metabolites to their hosts (Nikoh et al., 2011; Kaiwa et al., 2014; Salem et al., 2014). In addition to the primary gut symbiotic bacteria, these stinkbugs are also infected with such facultative symbiotic bacteria as *Wolbachia*, *Sodalis*, *Rickettsia*, *Spiroplasma, Lariskella*, and others. Of these, extensive infection surveys have been conducted for *Wolbachia*, *Sodalis* and *Lariskella* (Kikuchi and Fukatsu, 2003; Matsuura et al., 2012; Hosokawa et al., 2015), whereas *Spiroplasma* and *Rickettsia* have been detected in a few stinkbug species (Kikuchi et al., 2009; Caspi-Fluger et al., 2014; Matsuura et al., 2014; Amiri et al., 2020; Dally et al., 2020).

In this study, we surveyed diverse stinkbugs representing 13 families, 69 genera, 97 species and 468 individuals for *Spiroplasma* infection, thereby elucidating the infection frequency and the diversity of *Spiroplasma* associated with the stinkbugs. The *Spiroplasma*-positive stinkbug samples were subjected to molecular phylogenetic analysis and amplicon sequencing analysis targeting bacterial 16S rRNA gene, which uncovered sporadic and facultative nature of the stinkbug-*Spiroplasma* associations in general. In a stinkbug species that exhibited frequent *Spiroplasma* infection in natural populations, we investigated infection prevalence, vertical transmission and *in vivo* localization of *Spiroplasma* in detail.

## Materials and methods

### Insect materials

The stinkbug samples examined in this study are listed (Supplementary Tables S1, S2). Laboratory strains of the pea aphid *Acyrthosiphon pisum* (*Spiroplasma* negative) and the fruit fly *Drosophila melanogaster* (*Spiroplasma* positive and negative) were used as negative and positive control samples for *Spiroplasma* detection (Supplementary Table S3). The stinkbugs were collected in the field, of which some were preserved in acetone or 99% ethanol, some were preserved in ultracold freezers at –80°C, and others were freshly dissected in phosphate buffered saline (PBS) under a dissection microscope using fine forceps and razor blades. The white-spotted stinkbug *Eysarcoris ventralis* was maintained on sunflower seeds and water containing 0.05% ascorbic acid at 25°C under a long-day condition of 16 h light and 8 h dark.

### DNA sample preparation

After surface sterilization with 70% ethanol, the stinkbug samples were individually subjected to DNA extraction using QIAamp DNA mini kit (Qiagen). For relatively large insects (larger than 5 mm in size), the dissected alimentary tract was subjected to DNA preparation, whereas for relatively small insects (about 5 mm or smaller in size), the whole body was subjected to DNA preparation. For each sample, the dissected alimentary tract or the whole body was homogenized and digested in a lysis buffer containing proteinase K, purified using a spin column, and recovered with an elution buffer. For the initial proteinase K digestion, 200 μl of lysis buffer was used for most samples. For some samples large in size, 1,000 μl of lysis buffer was used for digestion and an aliquot (100–200 μl depending on the tissue size) was applied to the spin column for purification. The DNA samples were recovered with 200 μl of elution buffer, which were used as PCR templates without further dilution.

### Diagnostic PCR screening of *Spiroplasma*

Diagnostic PCR specifically targeting 16S rRNA gene of *Spiroplasma* was performed with the primers 16SA1 (5′-AGA GTT TGA TCM TGG CTC AG-3′; Fukatsu and Nikoh, 1998) and SpR5 (5′- CTG CAG CAC CGA ACT TAG TC-3′; Bastian and Foster, 2001) under the temperature profile of 98°C for 1 min followed by 30 cycles of 98°C for 10 s, 55°C for 15 s and 68°C for 45 s.

### Sequencing of 16S rRNA gene of *Spiroplasma*

For sequencing of 1.5 kb region of *Spiroplasma* 16S rRNA gene, in addition to the 0.8 kb region amplified by the primers 16SA1 and SpR5, a partially overlapping 0.9 kb region amplified by the primers SpF (5′- GCG CAG ACG GTT TAA CAA G-3′; Alexeeva et al., 2006) and 16SB1 (5’-TAC GGY TAC CTT GTT ACG ACT T-3′; Fukatsu and Nikoh, 1998) was directly sequenced as described previously (Kakizawa and Kamagata, 2014). The nucleotide sequences determined in this study were deposited in the DNA Data Bank of Japan^1^ under accession numbers LC685076- LC685096 (Supplementary Table S1).

### Molecular phylogenetic analysis

Multiple alignments were generated by the program Clustal Omega (Sievers et al., 2011). Ambiguously aligned nucleotide sites were manually removed. Phylogenetic analyses were conducted by maximum likelihood and neighbor-joining methods. The nucleotide substitution models were selected using MEGA version X (Kumar et al., 2018). Maximum likelihood trees and neighbor-joining trees were constructed by using MEGA version X. Bootstrap values were obtained with 1,000 replications.

### Amplicon sequencing analysis

The stinkbugs and other insect samples subjected to amplicon sequencing of bacterial 16S rRNA gene are listed (Supplementary Tables S3, S4). The V3-V4 region of bacterial 16S rRNA gene was amplified by the 2-step tailed PCR method as follows. The 1st PCR was performed with the primers 1st-341f_MIX (5′-ACA CTC TTT CCC TAC ACG ACG CTC TTC CGA TCT -NNN NN-C CTA CGG GNG GCW GCA G-3′; Muyzer et al., 1993) and 1st-805r_MIX (5′-GTG ACT GGA GTT CAG ACG TGT GCT CTT CCG ATC T-NN NNN- GAC TAC HVG GGT ATC TAA TCC-3′; Herlemann et al., 2011) under the temperature profile of 94°C for 2 min followed by 30 cycles of 94°C for 30 s, 50°C for 30 s and 72°C for 30 s, followed by 72°C for 5 min. PCR was performed with either ExTaqHS polymerase (TaKaRa) or TksGflex polymerase (TaKaRa). PCR products were purified with AMPure XP magnetic beads (BECKMAN COULTER). The 2nd PCR was performed with the primers 2ndF (5′-AAT GAT ACG GCG ACC ACC GAG ATC TAC AC -Index2- ACA CTC TTT CCC TAC ACG ACG C-3′) and 2ndR (5′-CAA GCA GAA GAC GGC ATA CGA GAT -Index1- GTG ACT GGA GTT CAG ACG TGT G-3′) using ExTaqHS polymerase under the temperature profile of 94°C for 2 min followed by 10 cycles of 94°C for 30 s, 60°C for 30 s and 72°C for 30 s, followed by 72°C for 5 min. Index1 and Index2 sequences were according to the Nextera XT Index Kit (Illumina). The 2nd PCR was for adding sequence indexes and tags for Illumina sequencing, and the cycles were minimized to reduce PCR-derived biases. The PCR products were purified with AMPure XP. Quantity of the library was analyzed by Synergy H1 with QuantiFluor dsDNA System (BioTek), and quality of the library was analyzed by Fragment Analyzer with dsDNA 915 Reagent Kit (Advanced Analytical Technologies). Finally, the library was sequenced on an Illumina MiSeq system with MiSeq Reagent Kit v3 (Illumina) that generated 2 × 300 bp paired end reads. The sequence data were analyzed as follows. First, the reads matching to the primer sequences were extracted by fastx_barcode_splitter tool of FASTX-Toolkit (ver. 0.0.14). The primer sequences were removed from all the reads by fastx_trimer of FASTX-Toolkit. Low quality reads were removed using sickle (ver. 1.33) based on the quality score 20, and reads shorter than 130 bp were also removed. All paired reads were combined using FLASH (ver.1.2.11) script based on >10-bp overlapping criteria. Subsequently, chimeric sequences were removed using the dada2 plugin of QIIME2 (ver.2021.4). In QIIME2, the chimeric-filtered sequences were clustered into operational taxonomic units (OTUs) using EzBioCloud 16S database.^2^ The OTUs were tabulated on each taxonomic level from phylum to genus and their relative abundances were calculated using a workflow script in QIIME2. All the above procedures were performed by Bioengineering Lab. Co., Ltd. (Kanagawa, Japan).

### Fluorescence *in situ* hybridization

Whole-mount fluorescence *in situ* hybridization (FISH) targeting bacterial 16S rRNA of *Spiroplasma* symbiont was performed as described previously (Matsuura et al., 2014). The dissected insect tissues were fixed in 4% paraformaldehyde solution in PBS for 3 h and then thoroughly washed three times in PBST (0.2% Tween20 in PBS). The fixed insect tissues were washed twice in a hybridization buffer (20 mM Tris–HCl, 0.9 M NaCl, 0.01% SDS, 30% formamide). For detection of *Spiroplasma* symbiont, the probe Spr403 (5′- AlexaFluor 555 - TAC TTA CTG TTC TTC CCT TAC A-3′; Matsuura et al., 2014) was used. Samples were incubated overnight at room temperature in the hybridization buffer containing 50 nM of the probe. After washed twice in PBST, nuclear DNA were stained with 4.5 μM of 4′,6-diamidino-2-phenylindole (DAPI; Thermo Fisher Scientific) for 1 h at room temperature, and then washed with PBT again (Koga et al., 2012). Samples were washed twice in PBST and mounted in 50% glycerol solution in PBS and observe a laser scanning confocal microscope (LSM 700, Carl Zeiss, Germany).

## Results

### Screening of *Spiroplasma* infection among diverse stinkbugs

Our large collection of stinkbug samples, consisting of 13 families, 69 genera, 97 species and 468 individuals (Supplementary Table S1), were subjected to diagnostic PCR detection of *Spiroplasma* infection. Of these, 4 families (30.8%), 7 genera (10.1%), 11 species (11.3%) and 21 individuals (4.5%) were diagnosed as *Spiroplasma* positive (Table 1). The following species contained *Spiroplasma* positive samples: *Riptortus pedestris* (1/1, Alydidae); *Acanthosoma denticaudum* (1/1, Acanthosomatidae); *Acanthosoma forficula* (1/1, Acanthosomatidae); *Acanthosoma labiduroides* (1/1, Acanthosomatidae); *Acanthosoma spinicolle* (3/3, Acanthosomatidae); *Adomerus triguttulus* (3/11, Cydnidae); *Macroscytus japonensis* (2/7, Cydnidae); *Eurydema dominulus* (1/10, Pentatomidae); *Eurydema rugosa* (1/17, Pentatomidae); *Eysarcoris ventralis* (6/7, Pentatomidae); and *Gonopsis affinis* (1/4, Pentatomidae) (Supplementary Table S1). These results suggested that, although not conclusive due to limited sample sizes, the *Spiroplasma* symbionts are, in general, facultative microbial associates of the stinkbugs.

**Table 1:** Summary of *Spiroplasma* detection from diverse stinkbugs representing 4 superfamilies, 13 families, 69 genera, 97 species and 468 individuals.

**Superfamily**	**Family**	**Genus**	**Species**	**Individual**
		*Spiroplasma*-positive taxa/taxa examined		
Coreoidea	Alydidae	1/1	1/1	1/1
	Coreidae	0/6	0/6	0/10
Lygaeoidea	Lygaeidae	0/1	0/1	0/1
	Rhyparochromidae	0/1	0/1	0/2
Pyrrhocoroidea	Pyrrhocoridae	0/1	0/1	0/1
Pentatomoidea	Acanthosomatidae	1/4	4/10	6/25
	Cydnidae	2/3	2/5	5/31
	Dinidoridae	0/1	0/1	0/17
	Parastrachiidae	0/1	0/1	0/6
	Pentatomidae	3/38	4/53	9/297
	Plataspidae	0/2	0/4	0/6
	Scutelleridae	0/8	0/10	0/64
	Urostylididae	0/2	0/3	0/7
Total		7/69	11/97	21/468

For detail, see Supplementary Table S1.

### Phylogenetic placement of *Spiroplasma* strains detected from diverse stinkbugs

**Figure 1:**
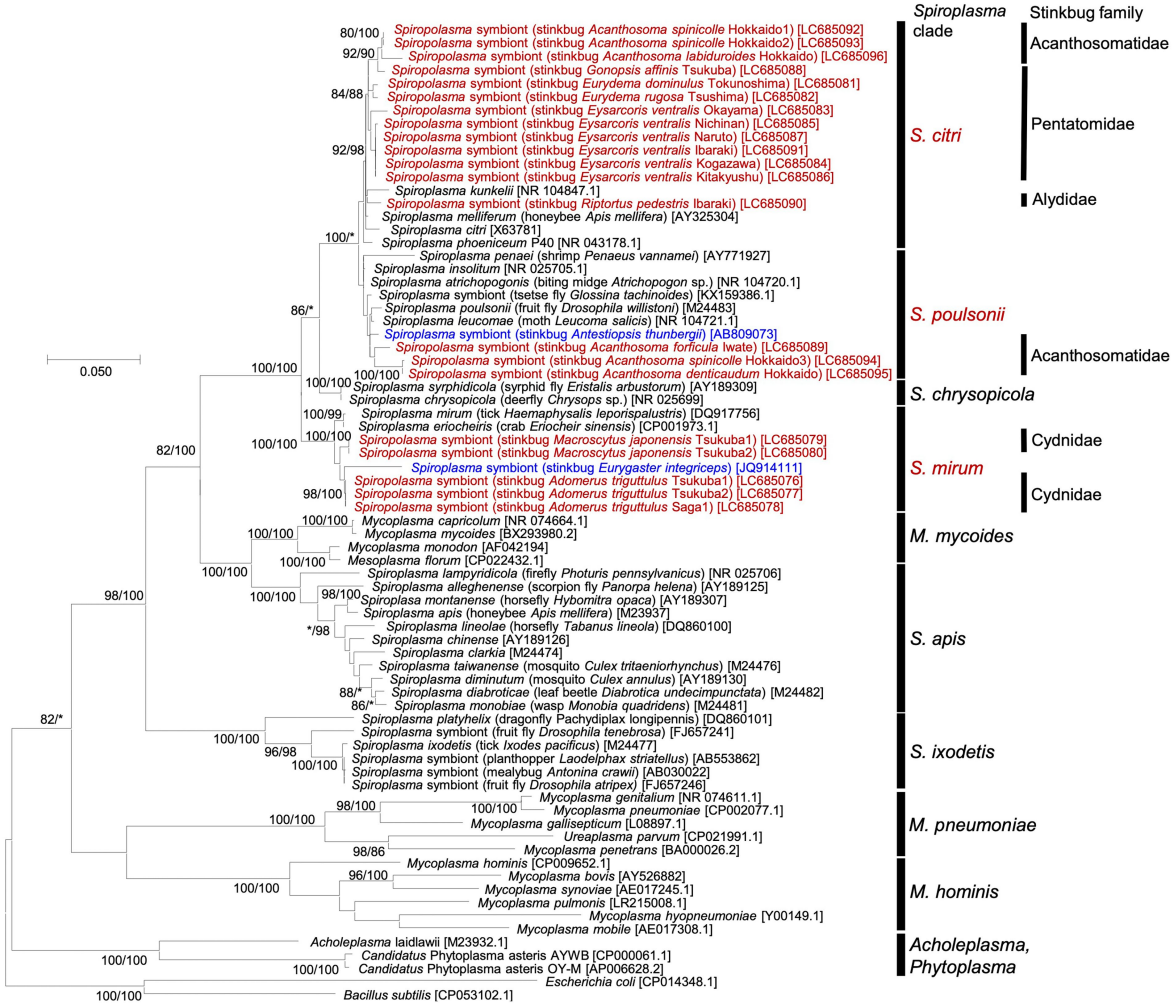
Phylogenic relationship of *Spiroplasma* symbionts detected from diverse stinkbugs based on 16S rRNA gene sequences. A maximum-likelihood phylogeny inferred from 1,632 aligned nucleotide sites is shown. Bootstrap support probabilities were obtained from 1,000 resamplings, of which the values of 80% or higher are shown at the nodes in the order of maximum likelihood/neighbor joining. Asterisks indicate support values lower than 80%. For each bacterial sequence, host-related information in parentheses and accession number in brackets are shown. The *Spiroplasma* sequences detected from stinkbugs in this study are highlighted in red, the *Spiroplasma* sequences detected from stinkbugs in previous studies (Matsuura et al., 2014; Amiri et al., 2020) are highlighted in blue, and the other sequences of *Spiroplasma* and allies retrieved from the databases are shown in black. The major *Spiroplasma* clades are depicted on the right side according to previously published papers (Paredes et al., 2015; Ballinger and Perlman, 2019) and the MolliGen database (Barré et al., 2004). On the right side, host stinkbug families for the *Spiroplasma* positive samples are also displayed.

All the 21 stinkbug samples positive of *Spiroplasma* infection were subjected to PCR amplification and sequencing of 1.5 kb region of 16S rRNA gene of *Spiroplasma*. The 16S rRNA gene sequences were subjected to molecular phylogenetic analysis together with *Spiroplasma* and allied bacterial sequences retrieved from GenBank and other DNA databases. The *Spiroplasma* symbionts detected from the stinkbugs were placed in either of the clades “*S. citri*,” “*S. poulsoni*” or “*S. minum*” (*sensu* Barré et al., 2004; Paredes et al., 2015; Ballinger and Perlman, 2019) in the family Spiroplasmataceae, the class Mollicutes, and the phylum Tenericutes (or Mycoplasmatota) (Figure 1). Note that *Spiroplasma* symbionts previously reported from stinkbugs also fell in these clades: the clade “*S. poulsoni*” for the coffee stinkbug *Antestiopsis thunbergii* (Pentatomidae) (Matsuura et al., 2014) and the clade “*S. mirum*” for the corn stinkbug *Eurygaster integriceps* (Scutelleridae) (Amiri et al., 2020). Globally, the phylogenetic relationship of the *Spiroplasma* symbionts did not reflect the phylogenetic relationship of the host stinkbugs. On the other hand, the *Spiroplasma* symbionts associated with the same stinkbug family tended to be related to each other: all the symbionts of cydnid stinkbugs were placed in the “*S. minum*” clade; almost all the symbionts of pentatomid stinkbugs were placed in the “*S. citri*” clade; and the symbionts of acanthosomatid stinkbugs were placed either in the “*S. poulsonii*” clade or the “*S. citri*” clade; (Figure 1). Furthermore, different individuals of the same stinkbug species tended to be associated with the same *Spiroplasma* symbiont as in *A. triguttulus*, *M. japonensis* and *E. ventralis*, although *A. spinicolle* represented an exceptional case (Figure 1).

### Microbiome of stinkbugs with and without *Spiroplasma* infection

**Figure 2:**
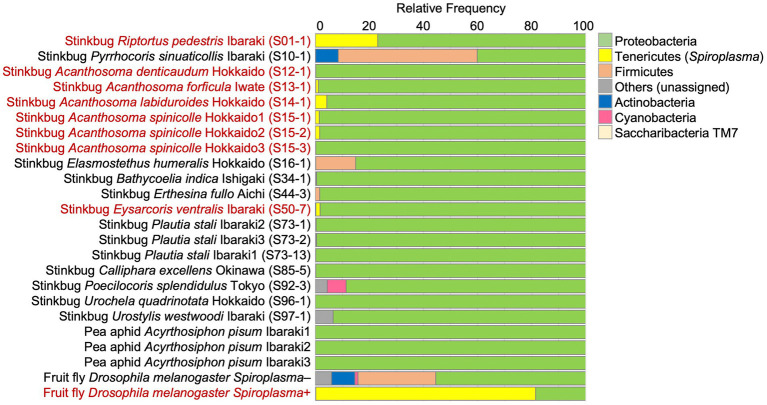
The relative abundance of the top seven bacterial phyla detected from stinkbugs and other insects by 16S rRNA gene amplicon sequencing. The phylum Tenericutes (or Mycoplasmatota) corresponds to *Spiroplasma.* The insect names in red indicate *Spiroplasma* positive ones. Sample numbers are shown in parentheses (Supplementary Table S1).

From 19 stinkbug samples, of which 8 and 11 were *Spiroplasma* positive and negative, respectively, the alimentary tracts were dissected and subjected to amplicon sequencing analysis of the V3-V4 region of bacterial 16S rRNA gene, with aphids and fruit flies as negative and positive control samples. *Spiroplasma* reads were specifically detected from the 8 *Spiroplasma* positive stinkbug samples (Figure 2; Supplementary Table S3), confirming the results of diagnostic PCR and molecular phylogenetic analysis (Supplementary Table S1; Figure 1). Reflecting the fact that stinkbugs generally harbor specific gut symbionts in their midgut symbiotic organ (Salem et al., 2015; Hosokawa et al., 2016; Takeshita and Kikuchi, 2017), the *Spiroplasma* reads generally occupied only a very small fraction of the total reads, ranging from 0.07 to 4.23%, with an exceptional case of 23.02% in *R. pedestris* (Figure 2; Supplementary Table S3). Phylum-, order- and family-level assignments of the reads verified that the majority of the reads were certainly derived from the gut symbionts of the stinkbugs: the Betaproteobacteria (= *Burkholderia*) dominant in *R. pedestris* (Coreoidea: Alydidae) (*cf.* Kikuchi et al., 2011); the Firmicutes (or Bacillota) (= *Clostridium*, *Lactococcus*) and the Actinobacteria (or Actinomycetota) (*Coriobacterium*, *Gordonibacter*) dominant in *Pyrrhocoris sinuaticollis* (Pyrrhocoroidea: Pyrrhocoridae) (*cf.* Sudakaran et al., 2012); and the Gammaproteobacteria (= Enterobacteriales) dominant in the other stinkbugs (Pentatomoidea: Acanthosomatidae, Pentatomidae, Scutelleridae and Urostylididae) (*cf.* Kikuchi et al., 2009; Kaiwa et al., 2014; Hosokawa et al., 2016, 2019) (Supplementary Tables S5–S7).

### Infection prevalence, vertical transmission, and *in vivo* localization of *Spiroplasma* in *Eysarcoris ventralis*

**Figure 3:**
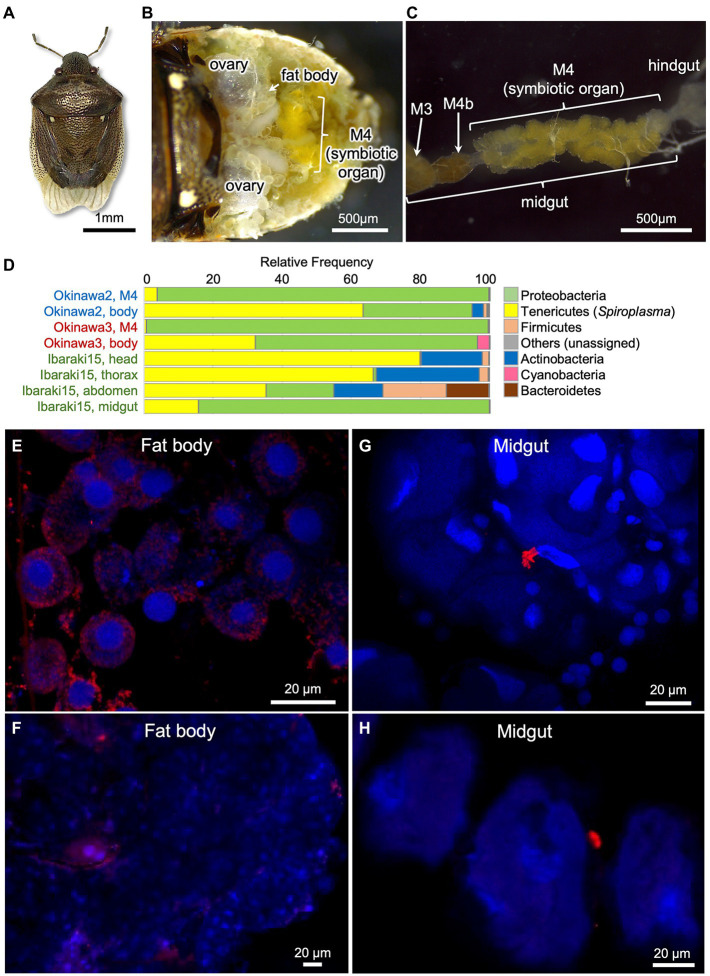
*Spiroplasma* symbiont associated with the white-spotted stinkbug *Eysarcoris ventralis*. **(A)** An adult insect. **(B)** Dissected abdomen. **(C)** Isolated alimentary tract. Abbreviations: M3, midgut third section; M4, midgut fourth section as the symbiotic organ; M4b, bulb-like section connected to M4 (see Oishi et al., 2019; Moriyama et al., 2022). **(D)** The relative abundance of the top seven bacterial phyla detected from three individuals of *E. ventralis* by 16S rRNA gene amplicon sequencing. The phylum Tenericutes (or Mycoplasmatota) corresponds to *Spiroplasma*. The sample names in the same color indicate those derived from the same insect individual. **(E–H)** FISH localization of *Spiroplasma* symbiont in fat body **(E,F)** and midgut symbiotic organ **(G,H)** of *E. ventralis*. **(E,F)** represent images derived from different individuals, so do **(G,H)**. Red signals represent *Spiroplasma* cells visualized by FISH whereas blue signals show insect nuclear DNA visualized by DAPI staining.

Among the 11 *Spiroplasma* positive stinkbug species, we focused on the white-spotted stinkbug *E. ventralis* (Figure 3A), because this species was maintainable on sunflower seeds in the laboratory at least for some period and exhibited a high *Spiroplasma* infection rate (6/7 = 85.7%) in our initial screening (Supplementary Table S1). We additionally collected 25 individuals of *E. ventralis* from 4 localities in Japan (Supplementary Table S2), and the insects were subjected to a series of experiments. Diagnostic PCR of dissected tissues and organs of *E. ventralis* (Figures 3B,C) identified a high *Spiroplasma* infection rate (22/25 = 88.0%; Supplementary Table S2). In some samples, *Spiroplasma* infection was not detected from the dissected midgut symbiotic organ but identified from the rest of the insect body, suggesting that *Spiroplasma* is mainly distributed among other tissues and organs than the midgut symbiotic organ (Supplementary Table S2). An adult female (= Ibaraki1; Supplementary Table S2) laid an egg mass in the laboratory, the hatchlings were reared on sunflower seeds, three newborn nymphs and three adults were subjected to diagnostic PCR, and all the insects were diagnosed as *Spiroplasma* positive (Supplementary Table S2). These results strongly suggested that *Spiroplasma* is passed to the next generation vertically in *E. ventralis*. We dissected three individuals of *E. ventralis* (= Okinawa2, Okinawa3 and Ibaraki15), and the dissected tissue samples were subjected to amplicon sequencing analysis of bacterial 16S rRNA gene. While the proteobacterial gut symbiont was predominant in the dissected midgut samples, *Spiroplasma* was dominant in all the other tissue samples (Figure 3D; Supplementary Tables S8–S10), suggesting that *Spiroplasma* infection is found throughout the body parts of *E. ventralis*.

Finally, we additionally collected five adult insects of *E. ventralis*, and their dissected tissues were subjected to FISH visualization of *Spiroplasma* cells (Supplementary Table S2). FISH signals were detected from all the insects, but density of the signals varied among individuals. In the fat body, intracellular signals were consistently detected, although distribution and density of the signals were quite variable (Figures 3E,F). In the midgut symbiotic organ, intracellular signals were scarcely observed, whereas sporadic signals outside the crypts were occasionally found, which might represent *Spiroplasma* cells in the hemolymph (Figures 3G,H).

## Discussion

In this study, we surveyed diverse stinkbugs representing 13 families, 69 genera, 97 species and 468 individuals for *Spiroplasma* symbionts. Thus far, *Spiroplasma* infections have been detected from diverse insects and other arthropods, plants, and some marine invertebrates (Anbutsu and Fukatsu, 2011; Ballinger and Perlman, 2019; Gasparich et al., 2020), but large-scale data of infection prevalence in natural host populations have been reported only in a few cases. To our knowledge, 10/136 (7.4%) species and 76/2052 (3.7%) individuals of diverse arthropods (Duron et al., 2008), 6/19 (31.6%) species and 284/2907 (9.8%) individuals of fruit flies (Watts et al., 2009), and 1/21 (11.3%) species and 24/566 (4.2%) individuals of aphids (Romanov et al., 2020) represent such reports. In comparison with these previous studies, our results that 11/97 (11.3%) species and 21/468 (4.2%) individuals of stinkbugs were *Spiroplasma* positive (Table 1; Supplementary Table S1) seem to present similar and relatively low infection rates, supporting the notion that the *Spiroplasma* symbionts are facultative microbial associates of insects and other arthropods in general.

Here it should be noted that the low detection rates of *Spiroplasma* in field-collected stinkbugs may entail under-estimation for the following reasons. (i) Limited sample size: Since a limited number of samples, often a single specimen, were examined for each host species, *Spiroplasma* infections at low levels in natural host populations may be frequently overlooked. (ii) Fluctuating symbiont density: Facultative symbionts generally exhibit relatively low infection densities, the infection densities may drastically vary depending on environmental conditions, and thus samples with very low infection density may be diagnosed as *Spiroplasma* negative. (iii) PCR efficiency, specificity and sensitivity: Primer and amplicon sizes may affect PCR efficiency, specificity and sensitivity. We note that the amplicon size of diagnostic PCR, 0.8 kb, adopted in this study is relatively large, which may be less efficient for *Spiroplasma* detection in comparison with 0.2–0.5 kb amplicons that are commonly used for diagnostic PCR. In contrast to the possibility of false negatives, all the *Spiroplasma* infections detected from 21 stinkbug species in this study are true positives on the ground that they were confirmed by gene sequencing.

Amplicon sequencing of bacterial 16S rRNA gene revealed that the *Spiroplasma* symbionts are quantitatively minor in comparison with the primary gut symbionts (Figure 2), confirming the notion that the *Spiroplasma* symbionts are facultative microbial associates of relatively low infection densities in the stinkbugs. On the other hand, in dissected tissues and body parts containing no midgut symbiotic organ, the *Spiroplasma* symbionts were detected as a major bacterial component (Figure 3D). The apparent *Spiroplasma* predominance in the dissected tissues is likely due to the absence of other bacteria rather than high *Spiroplasma* density in these tissues. In combination with the FISH observations (Figures 3E–H), these results suggest that the *Spiroplasma* symbionts are distributed in various tissues and organs at relatively low densities, at least in *E. ventralis*, like many other facultative symbiotic bacteria of insects and other arthropods (Oliver et al., 2010; Feldhaar, 2011).

Molecular phylogenetic analysis uncovered that the stinkbug-associated *Spiroplasma* symbionts are placed in at least three distinct clades in the Spiroplasmataceae (Figure 1), highlighting multiple evolutionary origins of the stinkbug-*Spiroplasma* associations. Obviously, the *Spiroplasma* phylogeny does not reflect the host stinkbug phylogeny (Figure 1), indicating the absence of host-symbiont co-speciation. On the other hand, the *Spiroplasma* symbionts associated with the same stinkbug family tend to be phylogenetically related to each other (ex. “*S. mirum*” with Cydnidae, “*S. poulsonii*” with Acanthosomatidae; see Figure 1). These patterns can be accounted for by the following ecological, physiological and/or evolutionary processes. (i) Physiological host specificity: Closely-related host insects may provide similar intra-host conditions for symbiotic bacteria, which may promote the preferential association of specific symbiont genotypes to specific host taxa. (ii) Ecological symbiont sharing mediated by horizontal symbiont transfers *via* common host plants: Closely-related host insects tend to use the same group of food plants, and plant-mediated symbiont transfers may cause the preferential association of specific symbiont genotypes to specific host taxa. It should be noted that plant-pathogenic *Spiroplasma* species are generally vectored by plant-sucking hemipteran insects (Gasparich et al., 2020) and some facultative symbionts of whiteflies and leafhoppers, such as *Rickettsia*, *Cardinium* and *Wolbachia*, have been reported to be horizontally transmitted *via* plants (Caspi-Fluger et al., 2014; Chrostek et al., 2017). (iii) Ecological symbiont sharing mediated by horizontal symbiont transfers *via* common parasites: Closely-related host insects tend to be exploited by common parasites, and parasite-mediated symbiont transfers may result in the preferential association of specific symbiont genotypes to specific host taxa. Previous studies reported that parasitoid wasps and blood-sucking mites may mediate such horizontal symbiont transmission (Jaenike et al., 2007; Gehrer and Vorburger, 2012). In fact, the evolutionary dynamics of the stinkbug-*Spiroplasma* associations seems likely to have been shaped by combination of these processes, which should be pursued in more depth in the future.

The large-scale infection prevalence data of *Spiroplasma* in stinkbugs are of particular interest in that such data have been also collected for other facultative symbionts, *Wolbachia* and *Sodalis*, in diverse stinkbugs (Kikuchi and Fukatsu, 2003; Hosokawa et al., 2015), which provide an opportunity to examine how different facultative symbiotic bacteria exhibit their specific infection patterns in the same host insect group. The infection frequency patterns are largely similar across the distinct symbiotic bacteria, *Wolbachia*, *Sodalis* and *Spiroplasma*: most stinkbug species were symbiont negative, some species exhibited intermediate infection frequencies, and several species showed 100% symbiont infection (Supplementary Figure S1). The predominance of the symbiont-free stinkbug species strongly suggests that the facultative symbiotic bacteria are generally not essential for the host stinkbugs. The intermediate infection frequencies may be realized by symbiont-mediated cytoplasmic incompatibility as known for many *Wolbachia* strains (Werren et al., 2008; Kaur et al., 2021), by symbiont-mediated defense against parasites and/or pathogens as known for some *Wolbachia* and *Spiroplasma* strains (Flórez et al., 2015; Ballinger and Perlman, 2019), or by symbiont-mediated fitness facilitation as known for a variety of facultative symbiotic bacteria (Oliver et al., 2010; Zug and Hammerstein, 2015). The fixed symbiont infections in a limited number of host species may have been maintained by symbiont-induced cytoplasmic incompatibility as known for diverse *Wolbachia* strains (Werren et al., 2008; Kaur et al., 2021), or by fitness improvement *via* nutritional supplementation as known for *Wolbachia* of bedbugs (Hosokawa et al., 2010; Nikoh et al., 2014; Moriyama et al., 2015) and *Sodalis pierantonius* in grain weevils (Oakeson et al., 2014; Vigneron et al., 2014). What mechanisms underpin the 100% infections with *Wolbachia* and *Sodalis* in several stinkbug species (Supplementary Figures S1A,B) is currently unknown and to be examined in future studies. Notably, no stinkbug species exhibited 100% infection with *Spiroplasma* in this study (Supplementary Figure S1C). To our knowledge, no *Spiroplasma* symbionts have been reported to exhibit 100% infection rates in natural arthropod populations (Duron et al., 2008; Watts et al., 2009; Anbutsu and Fukatsu, 2011; Romanov et al., 2020). These observations suggest the possibility that, although speculative, *Spiroplasma* might be inherently unlikely to evolve essential mutualism with arthropod hosts. This may be relevant to the fact that the genomes of *Spiroplasma* and allied bacteria, constituting the class Mollicutes, are highly reduced ancestrally, being devoid of most of the metabolic genes needed for establishment of nutritional mutualism *via* synthesis of amino acids and vitamins (Lo et al., 2016). On the other hand, some *Spiroplasma* genomes contain a number of laterally transferred genes (Lo et al., 2015), evidencing existence of a potential route for acquisition of such metabolic genes. Since our survey of *Spiroplasma* diversity has been quite limited in comparison with the enormous arthropod diversity in nature, it is conceivable that 100% infection cases might be discovered in wider surveys in the future.

In conclusion, we present the distribution and the diversity of the stinkbug-*Spiroplasma* symbiotic associations, thereby laying the foundation for future studies on the stinkbug-*Spiroplasma* symbiosis. The next step will be the establishment of an experimentally tractable stinkbug-*Spiroplasma* model system that is maintainable in the laboratory. In this study, we tried to establish a laboratory rearing system for *E. ventralis* that exhibited frequent *Spiroplasma* infections in natural populations, but it was not successful: fed with sunflower seeds and water supplemented with ascorbic acid, adult insects of *E. ventralis* survived for over a month, but produced few eggs and finally died out. Only a female managed to produce an egg mass, and emergence of *Spiroplasma*-infected offspring from the eggs strongly suggested vertical transmission of the *Spiroplasma* symbiont, but more data are needed to verify this idea. In this context, we regard the bean bug *Riptortus pedestris* as a promising model system, on the grounds that *R. pedestris* is easily and stably maintainable in the laboratory and widely used for studies on the gut symbiotic bacteria of the genus *Burkholderia* (Takeshita and Kikuchi, 2017; Kaltenpoth and Flórez, 2020). In this study, we were able to examine only a field-collected sample of *R. pedestris*, in which the *Spiroplasma* infection density was remarkably high (Figure 2). Thus far, we have inspected several laboratory strains of *R. pedestris*, but unfortunately, they were all free of *Spiroplasma* infection. However, we expect that a wide field survey in the next season will lead to the establishment of *Spiroplasma*-infected laboratory strains of *R. pedestris*, which will enable us to experimentally investigate important biological aspects of the stinkbug-*Spiroplasma* symbiotic association such as fitness effects, reproductive phenotypes, vertical transmission route and efficiency, etc.

## Data availability statement

The datasets presented in this study can be found in online repositories. The names of the repository/repositories and accession number(s) can be found at: https://www.ddbj.nig.ac.jp/, LC685076-LC685096; https://www.ddbj.nig.ac.jp/, DRR358028-DRR358051; https://www.ddbj.nig.ac.jp/, DRR359516-DRR359523.

## Author contributions

SK, TH, and TF: conceived the study. TH, KO, and TF: collected the stinkbug samples. SK, TH, and KM: performed diagnostic PCR screening and 16S rRNA gene sequencing. SK and TH: conducted molecular phylogenetic analysis. SK and KM: performed amplicon sequencing analysis. KO: conducted FISH. TF and SK: wrote the manuscript. All authors contributed to the article and approved the submitted version.

## Funding

This study was supported by the JST ERATO grant number JPMJER1902 to SK and TF, and the JSPS KAKENHI grant number JP17H06388 to TF. KO was supported by the JSPS research fellowships for young scientists (21J01321 to KO).

## Conflict of interest

The authors declare that the research was conducted in the absence of any commercial or financial relationships that could be construed as a potential conflict of interest.

## Publisher’s note

All claims expressed in this article are solely those of the authors and do not necessarily represent those of their affiliated organizations, or those of the publisher, the editors and the reviewers. Any product that may be evaluated in this article, or claim that may be made by its manufacturer, is not guaranteed or endorsed by the publisher.

## Footnotes

^1^ http://www.ddbj.nig.ac.jp/index-e.html

^2^ https://www.ezbiocloud.net
