# Supplementary figures and images for: Spiroplasma as facultative bacterial symbionts of stinkbugs
Source: Front Microbiol. 2022 Oct 24;13:1044771. doi: 10.3389/fmicb.2022.1044771 (PMC9638005; doi:10.3389/fmicb.2022.1044771)

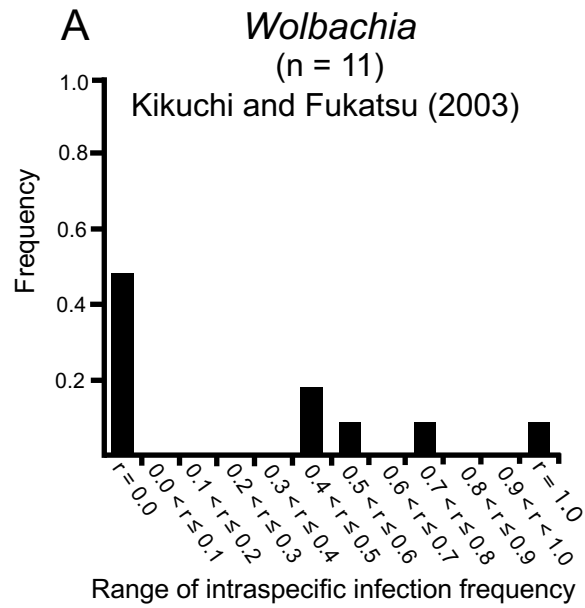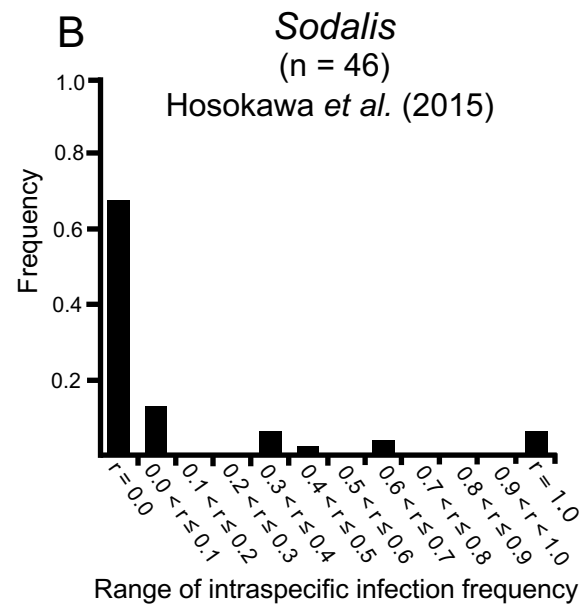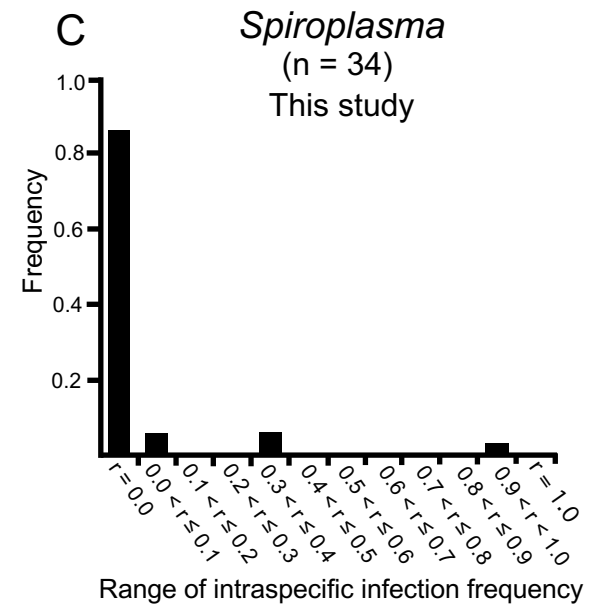

Supplement: Supplementary file 1 [file Data_Sheet_1.zip › Figure S1.PDF]
